# Supplementary material for: Germline polymorphisms in the immunoglobulin kappa and lambda loci underpinning antibody light chain repertoire variability
Source: Nat Commun. 2025 Nov 28;16:11707. doi: 10.1038/s41467-025-66759-9 (PMC12753720; doi:10.1038/s41467-025-66759-9)
Supplement: Supplementary file 3 — Description of Additional Supplementary Files [file 41467_2025_66759_MOESM3_ESM.pdf]

Description of Additional Supplementary Files

File Name: Supplementary Data 1
Description: Sample information with AIRR-seq and Pacific BioScience sequencing details

File Name: Supplementary Data 2
Description: SV allele frequencies and number of samples with at least one SV allele

File Name: Supplementary Data 3
Description: IGK alleles identified in all individuals with PacBio sequencing (n = 177)

File Name: Supplementary Data 4
Description: IGL alleles identified in all individuals with PacBio sequencing (n = 177)

File Name: Supplementary Data 5
Description: Samples for which each allele of an IGKV proximal paralog was distinct from the sequence of each allele of the distal paralog

File Name: Supplementary Data 6
Description: Unmutated and mutated repertoire guQTL statistics from linear regression (Bonferroni corrections: IGK; P < 3.7e-5, IGL; P < 1.9e-5)

File Name: Supplementary Data 7
Description: Comparison of unmutated and mutated repertoire guQTL statistics from linear regression (Bonferroni corrections: IGK; P < 3.7e-5, IGL; P < 1.9e-5)

File Name: Supplementary Data 8
Description: Fisher exact test (one-sided) for enrichment of guQTLs in TFBS

File Name: Supplementary Data 9
Description: LD blocks in IGK and IGL

File Name: Supplementary Data 10
Description: Overlap of genes and guQTLs in LD blocks

File Name: Supplementary Data 11
Description: Fisher exact test (two-way) of allelic variation among individuals in genotype groups at lead guQTLs

File Name: Supplementary Data 12
Description: Unmutated and mutated repertoire CDR3 property QTL statistics (significant variants)
